# The Neural Correlates of Identity Faking and Concealment: An fMRI Study

**DOI:** 10.1371/journal.pone.0048639

**Published:** 2012-11-07

**Authors:** Xiao Pan Ding, Xiaoxia Du, Du Lei, Chao Super Hu, Genyue Fu, Guopeng Chen

**Affiliations:** 1 School of Psychology and Cognitive Science, East China Normal University, Shanghai, P.R. China; 2 Department of Psychology, Zhejiang Normal University, Hangzhou, P.R. China; 3 Department of Physics, Shanghai Key Laboratory of Magnetic Resonance, East China Normal Universtity, Shanghai, P.R. China; University of Regensburg, Germany

## Abstract

The neural basis of self and identity has received extensive research. However, most of these existing studies have focused on situations where the internal representation of the self is consistent with the external one. The present study used fMRI methodology to examine the neural correlates of two different types of identity conflict: identity faking and concealment. Participants were presented with a sequence of names and asked to either conceal their own identity or fake another one. The results revealed that the right insular cortex and bilaterally inferior frontal gyrus were more active for identity concealment compared to the control condition, whereas identity faking elicited a significantly larger percentage signal increase than the control condition in the right superior frontal gyrus, left calcarine, and right caudate. These results suggest that different neural systems associated with both identity processing and deception were involved in identity concealment and faking.

## Introduction

The question of who we are and who we are not is an important existential question. This question of self and personal identity is central to philosophy because it cuts across a host of important philosophical issues such as mind-body distinction, consciousness, and free will. Personal identity is also an important issue in psychology, especially in self-psychology [1], and it is concerned with a variety of issues such as self-recognition, self-awareness, self-concept, self-esteem and confidence, and self-referential processing.

Recently, the neural basis of self and identity has received extensive research [2]–[8]. Most of these existing studies have come to an agreement that the cortical midline structures (CMS) including the medial prefrontal cortex, the anterior cingulate cortex, and the precuneus, play an important role in processing information concerning the self, whereas the mirror neurons structures (MNS) seem to also be involved with self-recognition and related social understanding [8]. Further, many studies have shown that the processing of self information tends to be right lateralized [3], [7].

Most of the existing neural imaging studies on the self have focused on situations where the internal representation of the self is consistent with the external one. That is, participants are typically asked to process personal information about themselves that is truly corresponding to their actual self. However, little neural imaging research has been done to examine the neural correlates of personal identity faking and concealment where one’s internal representation of oneself may be in conflict with their external presentation. Although individuals in general are typically consistent in their internal and external identities, it is not uncommon that individuals may present themselves differently from their true identity (e.g., in situations of identity frauds, or clandestine operations). It is entirely unclear whether and to what extent the neural systems involved in the processing of the self identity in typical situations would also be engaged during such identity conflict.

The present study aimed to address this important gap in the literature. Specifically, we used fMRI methodology to examine the neural correlates of personal identity faking and concealment. Identity faking and concealment are common in everyday life. One of the important tasks of agents working for a clandestine operation is often to conceal their own identity and assume another identity. Some people may also conceal their own identity and fake another one for fraudulent and criminal reasons. The examination of the neural basis of identity faking and concealment allows for identifying not only brain regions involved in processing the self information in general, but also brain regions involved in identity conflict.

Although no study has examined the neural basis of identity faking and concealment, there has been a large body of related neural imaging studies. One is primarily focused on the neural basis of self-identity recognition, while the other investigates the neural mechanisms underlying deception. Regarding self-identity recognition, it should be noted that a person’s identity can be based on a host of information such as the person’s face, name, voice, body, and personality. A growing body of evidence has shown that the right fronto-parietal network plays an important part in self-recognition or self-other discrimination based on such information as faces or names [8]–[10]. For example, Perrin et al. (2005) found that the amplitude of the P3 component, elicited when hearing one’s own name, correlates with the regional cerebral blood changes in the right superior temporal sulcus, precuneus, and medial prefrontal cortex [11]. Carmody and Lewis (2006) used fMRI methodology and found greater activations in the middle frontal cortex, middle and superior temporal cortex, and cuneus in response to hearing one’s own first name in contrast to hearing the name of others [12]. Another fMRI study, which also compared responses to auditory presentations of own versus other’s first names, showed activations in the right paracingulate cortex, the right and left temporal cortex, as well as in the superior frontal gyrus (SFG) and the inferior frontal gyrus (IFG)/insula bilaterally [13]. Tacikowski et al. (2010) recently found that the self-name recognition is associated with robust activations in widely distributed bilateral neural networks including the fronto-temporal, limbic, and subcortical structures [14].

Regarding deception, there have been extensive neural imaging studies (for reviews, see [15], [16]), although none have examined the neural mechanisms associated with identity faking or concealment. Most of the existing studies typically asked participants to lie about recently learned knowledge such as words [17]–[20], cards [21]–[27], pictures [28], [29], faces [30], or numbers [18]. Participants have also been asked to lie about past autobiographic experiences, a situation pertinent to identity faking or concealment [18], [31]–[37]. Most of these studies found deception-related activities in the PFC and ACC [16], [18], [22], [24], [38]–[40]. The activations in these areas are not surprising because such findings are consistent with the conceptualization of deception as an executive control intensive task [24], [41]–[43]. To deceive, one must inhibit the public disclosure of the true state of affairs and present instead a false state of affairs publicly, which requires a host of executive functions such as inhibition, working memory, and planning [16], [44]–[46]. Miyake and colleagues [47] proposed that executive control may comprise three different component processes: 1) working memory, 2) task switching, and 3) inhibitory control. All 3 components of executive control may contribute to deception: suppressing a truthful response while reporting false information (inhibitory control); switching between truthful and deceptive responses (task switching); and keeping the details of the lie as well as the truth in mind in order to successfully maintain a lie (working memory) [16], [37], [41]. Christ et al. (2009) used an activation likelihood estimate (ALE) method of meta-analysis to identify overlapping regions between deception and executive control, and found that overlaps occurred in the bilateral anterior insula, left IFG, left middle frontal gyrus, right intermediate frontal sulcus, right ACC, and right intraparietal sulcus.

Building on the existing neural imaging studies of the self in non-deceptive situations, and those of deception about recently learned or autobiographic information, the present study investigated the neural correlates of identity faking and concealment and the similarities and differences between the two. Self-identity is a broad concept that covers a variety of aspects including: name, birth place, gender, nationality, language and so on. In the present study, we chose to focus on self-name as the part of personal identity of interest. Personal name is one typical form of identity. It is not only the carrier of the personal identity which can be used for expressing self and distinguishing self from others, but also embodies the characteristic of social identity because it represents relationships in certain groups [48]. There is evidence suggesting that self-name is very closely attached to a person’s inner sense of identity. For example, the state of namelessness is considered equal to having no honor or identity in China [49]. In the present study, we asked participants to lie about their own personal identity. We presented participants with a sequence of names and asked them to either conceal their own identity or fake another one. Participants could conceal their identity by denying a presently seen name to be their true name even though it was the case. They could also fake an identity by claiming a presently seen name to be their true name even though it was not the case. Also, they were shown control names which they could truthfully deny to be their true name. To reveal neural correlates of identity faking and concealment, we compared the brain activations in the identity faking condition to those in the control condition, as well as the brain activations in the identity concealment condition to those in the control condition.

Based on the existing research reviewed above, we hypothesized that because identity concealment specifically calls for inhibition of one’s true personal identity, the identity concealment condition would produce greater activations in regions in the PFC, ACC, and right frontoparietal area. This hypothesis was derived specifically from findings that the PFC and ACC have been associated with inhibitory control during deception [18], [22], [24], [38]–[40], and the right frontoparietal area has been associated with self-recognition [8]–[10], [50]. Because identity faking requires one to generate a name differing from their true identity, working memory is thought to be specifically involved. Thus, we expected that the identity faking condition would specifically produce greater activations in the regions of DLPFC and CMS. DLPFC has been associated with working memory [16], and pretending to know [17], and the CMS has been consistently found to be associated with self-referential processing [4], [8], [51].

## Methods

### Ethics Statement

All procedures used in the current study were approved by the Ethics Committee of Zhejiang Normal University. All participants signed a written informed consent prior to their participation in the study. They were told that they had the option to quit at any time during the experiment and still receive monetary payment.

### Participants

Fourteen right-handed Chinese participants were recruited. Two of them were excluded due to errors in procedure or excessive head movements. Thus, there were 12 valid participants (6 females, 6 males, mean age: 25.4 years, SD = 4.17 years). All participants were screened to rule out head trauma, history of neurological or psychiatric disorders, substance abuse, or other serious medical conditions. None of them had ever changed their first or last name.

### Stimuli and Design

Full Chinese names (surname plus given name) in the form of Chinese characters were presented as BMP images on the computer monitor (640×480). There were four types of names. The first was the participant’s true name, which would be used in the identity concealment condition. The second was a name that did not belong to the participant, which was used in the identity faking condition. The third was a control name to be used in the control condition. In addition, there were 10 irrelevant names that were not used in any of the above conditions but were mixed with the above names in the sequence of stimulus presentation. In the three conditions, each type of name was presented 30 times. The irrelevant names were each presented 3 times and thus the irrelevant names were also presented 30 times in total.

**Figure 1:**
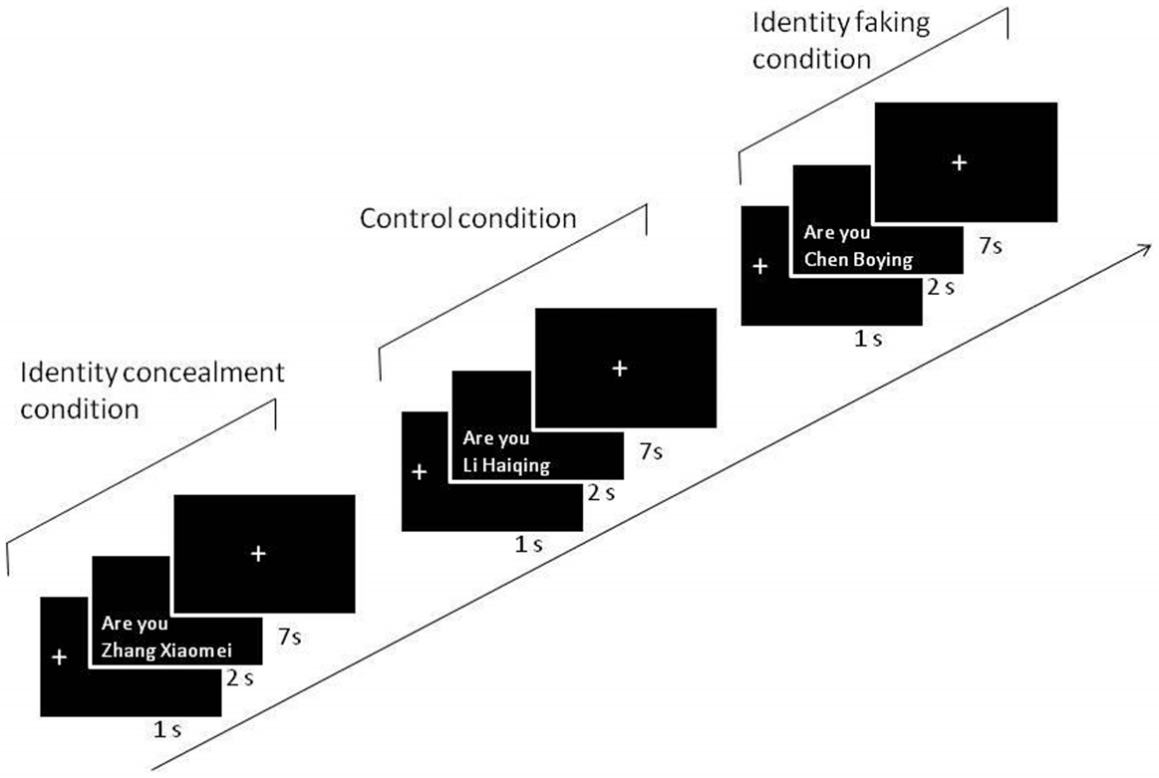
The schematic diagram of the experiment. (In the sample trial depicted here, the participant whose name was Zhang Xiaomei was asked to deny she was Zhang Xiaomei, but to admit falsely that she was Chen Boying. In this trial of the control condition, she was asked to respond correctly that she was not Li Haiqing.)

The whole experiment consisted of 120 trails. Each trial began with the presentation of the fixation (1 sec), followed by the name in the form of a question, ‘Are you XXXX (name)?’ (2 sec), after which the participant had to respond within 7 seconds. Participants should have pressed the ‘NO’ button to all of the names except for the fake name (see Figure 1). All of the names were presented in a pseudorandom order. The software package IFIS-SA was used to present stimuli and record responses.

**Table 1:** Means (standard deviations) of accuracy and response latency in the four conditions.

	Accuracy	Response latency (ms)
Identity concealment	97.22 (2.39)	785 (160)
Identity faking	94.44 (4.79)	805 (155)
Control condition	93.06 (5.41)	761 (134)
Irrelevant names	95.56(4.34)	801(191)

### Procedure

Before the imaging session, experimenter A told participants that this was a spy detection test and that they should try to protect their own identity and not be detected by the scanner. To do so, participants needed to deny their own names and acknowledge their assumed name in the imaging phase of the study. Each participant saw his/her list of names and made sure that the names on the list (except for his/her own name) were novel names and none were names of people they knew. Then, participants were required to draw one of three envelopes and were led to believe that each envelope contained different names that could be used as their fake name. In fact, each envelope contained the same, gender neutral name. The participants were instructed to memorize the name and pretend to use it as their own name throughout the entire experiment. They were also asked to conceal their own names. After they practiced for 20 trials, they were brought into the scanning room. This experimental procedure was modeled after that used extensively in previous studies that required participants to conceal certain pieces of information [21], [22], [24]–[26], [52]. After the imaging session, participants were debriefed and queried about their fake name to make sure the subjects really used the fake name they received before the experiment.

**Figure 2:**
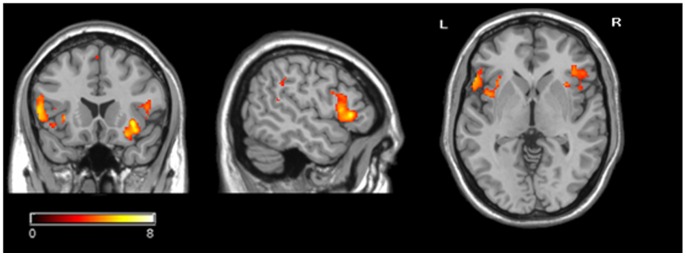
Brain areas showing a significant main effect of identity concealment (the identity concealment condition vs. the control condition) in the group analysis. Color scale represents t score values.

**Figure 3:**
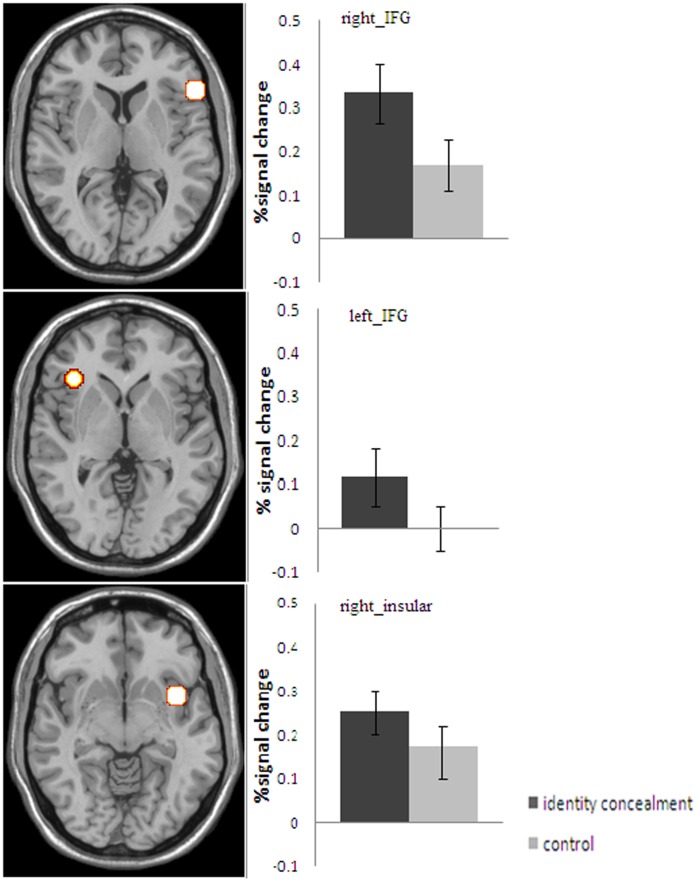
The mean percentage signal changes elicited by the identity concealment vs. the control condition.

**Table 2:** Regional brain activity differing between the identity concealment condition and the control condition.

Regions	Side	BA	Cluster	Peak voxel (MNI coordinate)			T	z
				X	Y	Z		
Inferior Frontal Gyrus	L	47	214	−38	26	−6	6.57	4.11
				−46	30	−14	6.22	3.99
				−34	18	−8	5.91	3.89
Inferior Frontal Gyrus	R	45	175	54	24	2	6.41	4.06
				56	14	2	5.23	3.63
				50	12	12	4.52	3.33
Insular cortex	R	13	81	38	8	−10	7.36	4.34

*Note*. Regions included in a single cluster are listed together. The spatial extent of each cluster was > = 20 voxels and an FWE of .05 was used to correct for multiple comparisons.

### fMRI Data Acquisition and Analysis

Functional imaging data was performed in a 3.0T Tim Trio system (Siemens Medical Systems Erlangen, Germany) using a twelve-channel head coil in the Shanghai Key Laboratory of Magnetic Resonance. In the experiment, a T2*-sensitive ultrafast multi slice echo planar imaging (EPI) sequence sensitive to blood oxygen level-dependent (BOLD) contrast was used. Thirty-three transversal slices of functional images that covered the whole brain were acquired using a single shot gradient echo EPI sequence with TR = 2000 ms, TE = 30 ms, flip angle 90°, matrix size = 64×64, slice thickness = 3 mm, field of view  = 220 mm^2^.

**Figure 4:**
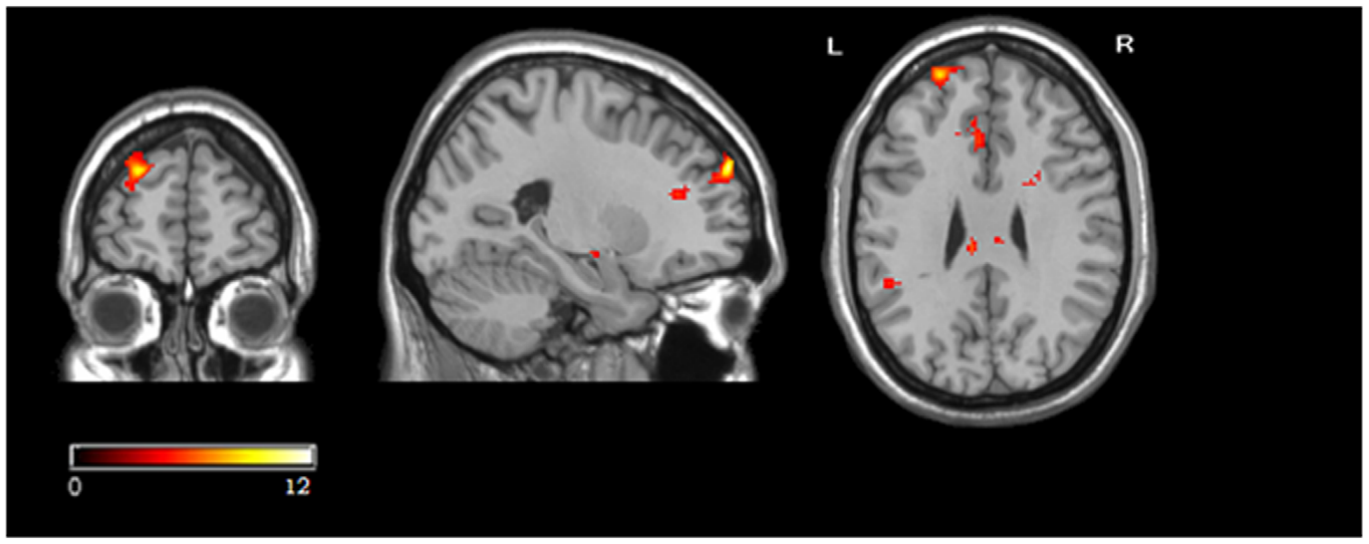
Brain areas showing a significant main effect of identity faking (the identity faking condition vs. the control condition) in the group analysis. Color scale represents t score values.

**Figure 5:**
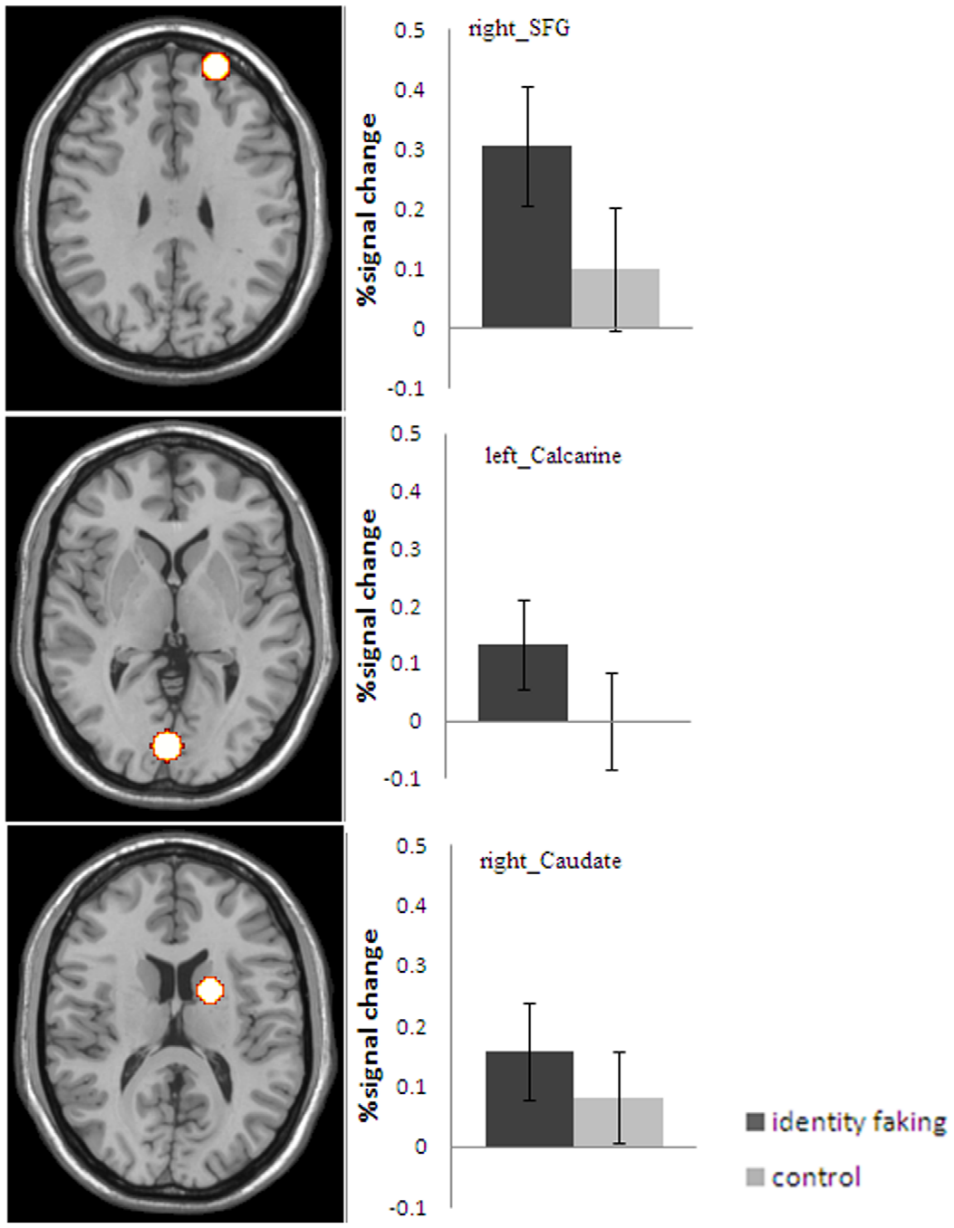
The mean percentage signal change elicited by the identity faking vs. the control condition.

**Table 3:** Regional brain activity differing between identity faking and control condition.

Regions	Side	BA	Cluster	Peak voxel (MNI coordinate)			T	z
				X	Y	Z		
Superior Frontal Gyrus	R	9/10	226	22	58	32	11.67	5.25
				30	52	16	6.14	3.96
				26	50	24	5.32	3.67
Calcarine	L	17/18	448	−4	−86	2	8.73	4.68
				−22	−88	0	7.99	4.51
				−14	−86	0	6.58	4.11
Caudate	R	32/24	747	18	2	18	8.63	4.66
				6	−14	8	7.10	4.26
				16	24	16	6.87	4.20

*Note*. Regions included in a single cluster are listed together. The spatial extent of each cluster was > = 20 voxels and an FWE of .05 was used to correct for multiple comparison.

The imaging data were analyzed using Statistical Parametric Mapping software (SPM8, Wellcome Department of Cognitive Neurology, London, UK. http://www.fil.ion.ucl.ac.uk/spm). The functional data of each participant were motion and slice-time corrected, spatially normalized into the standard MNI space, and smoothed using a Gaussian kernel of 8 mm FWHM. To obtain regions differentially activated by the different item types, we modeled the four item classes (identity concealment, identity faking, control condition, and irrelevant items) as separate regressors that were convolved with the canonical hemodynamic response function using a general linear model (GLM) at the individual level. Simple contrast maps (identity concealment vs. control condition, identity faking vs. control condition, identity concealment vs. identity faking) were then entered into a random effects analysis to identify regions that showed significant activation differences between item types (t-contrast). Post hoc analyses were accomplished by separate comparisons of the average percentage signal change as a function of item type in all regions of interest (ROI), functionally defined by the t-contrast of the aforementioned random effects analysis. To minimize the biased selection of voxels for individual differences regression analysis, the functionally defined ROIs were replaced with spherical ROIs (radius 8 mm) centered on the centers of mass of the original ROIs. These values were obtained using the MarsBaR (http://marsbar.sourceforge.net).

For all random-effects SPM-analyses, p-values were corrected for multiple comparisons using a FWE of .05. Additionally, activations were required to reach a spatial extend threshold of at least 20 contiguous voxels.

To distinguish the spatial patterns of brain activities between the concealment condition and the faking condition, we extracted the time course data for the ROIs from smoothed images. These features were then entered into a Support Vector Machine (SVM) with a non-linear kernel named radial basis function after using the principal component analysis according to the correlational matrix. We analyzed these data using the libsvm toolbox [53]. We used a cross validation method called the “Out” method. Using this method, data from 11 subjects were used to train the support vector machine and then the remaining one was used to be predicted by the SVM model. This procedure was repeated 12 times. The advantage of the “Out” method is that it controls for individual differences; however the size of training samples is not as big as other methods [54]. To further explore whether the brain activities in each ROI were different for the identity faking and concealment conditions, we performed the same SVM procedures on the data from each ROI.

## Results

### Behavioral Data

The means and standard deviations of participants’ accuracy and response latencies for each of the three conditions were shown in Table 1. Participants were generally highly accurate in faking identity, concealing their true identity, and rejecting the control name. A significant difference in accuracy was observed among the three conditions, *F*(2, 9) = 3.61, *p* = .044, *η* ^2^ = .25. A priori repeated contrasts revealed that this significant effect was due to the difference between the identity concealment and faking conditions (participants were slightly more accurate in the identity concealment condition, *F*(1, 10) = 7.86, *p* = .017, *η* ^2^ = .42). There were no significant differences in response latency among the three conditions (*F*(2, 9) = 1.99, *p* = .160, *η* ^2^ = .15).

### fMRI Data

#### Identity concealment vs. control

Whole brain analyses contrasting the identity concealment condition and the control condition revealed significant activation differences in the bilateral inferior frontal gyrus (IFG, BA47 and BA45) and the right insular cortex (BA13), FWE corrected, *p*<.05 (Table 2 & Figure 2). In the left IFG, right IFG, and right insular cortex, the concealed names elicited a significantly larger percentage signal increase than the control names, *t*(11) = 5.18, *p*<.001, *t*(11) = 6.10, *p*<.001, and *t*(11) = 4.23, *p* = 0.001, respectively (Figure 3).

#### Identity faking vs. control

Significant activation differences between the identity faking and control conditions were observed in the right superior frontal gyrus/DLPFC (BA9/10), the left calcarine (BA17/18) and caudate(BA32/24)(Table 3, Figure 4). In the right superior frontal gyrus, left calcarine, and caudate, the fake names elicited a significantly larger percentage signal increase than the control names, *t*(11) = 11.32, *p*<.001, *t*(11) = 6.62,*p*<.001, and *t*(11) = 6.69, *p*<.001, respectively (Figure 5).

#### Identity concealment vs. faking

Simple GLM contrasts failed to reveal any significant differences between the identity concealment condition and the faking condition in any brain regions. However, this null result does not necessarily suggest that there was no difference between the identity faking and concealment conditions. One possibility is that the two conditions may activate an overlaping but different networks of brain regions. Because these differences may be more sutble than the differences between the experimental conditions and the controls, the traditional GLM may not be able to uncover them. Further, the relatively small sample of the present study may also have prevented us from uncovering differential activation patterns produced by the two identity conditions. To test this possibility, we used the more sensitive SVM machine learning method. An added bonus of using this method is that it can provide information not only about whether the two idendity conditions produced differential activations in a particular ROI, but also whether the activation patterns among several ROIs were different between the two experimental conditions.

We thus extracted the time course data for six ROIs that were more positive to either identity faking or concealment relative to the control condition. Three ROIs were chosen from the identity concealment vs. control contrast (the right insula, right IFG and left IFG) and the others from the identity faking vs. control contrast (the right SFG, left calcarine and right caudate) in the above GLM analyses. Each ROI had a size of 8 mm×8 mm×8 mm with each voxel size being 2 mm×2 mm×2 mm, so there were 257 voxels in each ROI, resulting in 257×6 = 1562 values for the six ROIs. These 1562 values are henceforth referred to as features in the SVM machining learning methods. There were 30×12 = 360 samples for both the identity concealment condition and the identity faking condition. These features were entered into the SVM with a non-linear kernel called the radial basis function using the libsvm toolbox [53]. The “Out” method could reliably differentiate the brain activities in the six ROIs associated with either identity faking or identity concealment. The classification accuracy was 62.64% (signal detection discriminability d’ = .67). One sample test showed that the accuracy was significantly above chance (50%, or d’ = 0, *t*(11) = 5.501, *p*<.001).

To explore whether there were any activation pattern differences between the two experimental conditions in a particular ROI, the time course data from each ROI was entered in SVM with linear multinomial kernel analysis after using principal component analysis according to the correlational matrix. The results showed that each ROI (the right IFG, right insula, left calcarine, right caudate, and right SFG), except for the left IFG, could be used to differentiate the brain activities reliably when using the “Out” methods. The classification accuracies were 60.69% (d’ = .55) for the right IFG, 58.75% (d’ = .45) for the right insula, 58.75% (d’ = .45) for the left calcarine, 58.75%(d’ = .45) for the caudate, and 58.19%(d’ = .43) for the right SFG. All accuracies were significantly above chance (50%), *t*(11) = 7.34, *p*<.001, *t*(11) = 3.41, *p*<.01, *t*(11) = 4.65, *p*<.005, *t*(11) = 3.99, *p*<.005 and *t*(11) = 3.29, *p*<.01, respectively. Further inspection of the activation data showed that identity concealment produced greater activations than identity faking in the right IFG and the right insula, whereas identity faking produced greater activations in the left calcarine, SFG, and caudate.

In addition to machine learning methods, ROI analysis was also conducted using 6 ROIs that were more positive to either identity faking or concealment relative to the control condition. The result showed that fake names elicited a significantly larger percentage signal increase than the concealed names (*t*(11) = 2.68, *p*<.05) in the right SFG. However, the reversed contrast yielded no significant results.

## Discussion

The present study was the first attempt to investigate neural activities associated with identity conflict and deception. More specifically, we examined whether there existed similar or distinctive neural networks for processing fake versus concealed identities. Several major findings were obtained. First, the right insula and bilaterally inferior frontal gyrus were more active in the identity concealment condition than in the control condition. Second, identity faking elicited significantly larger activations than the control condition in the right superior frontal gyrus, left calcarine, and right caudate. Third, although the traditional GLM analyses failed to show any activation differences between identity faking and concealment, a more sensitive machine learning method (SVM) uncovered subtle but significant differences both in terms of patterns of activations in the above mentioned brain regions individually and in terms of patterns of activations between the brain regions. Thus, the present findings taken together suggest that identity conflict may engage a network of brain regions and the network may be different from that for processing identity information when no identity conflict exists. Furthermore, identity faking and concealment may engage overlapping yet somewhat different neural networks.

When compared to the existing findings in the literature, it seems that the neural networks for identity faking and concealment may be related to the self-network and the network for deception. Furthermore, the involvement of the two networks for identity faking and concealment was also not entirely identical.

### Identity Faking and Concealment in the Self-network

Regarding identity concealment, we found that the bilateral inferior frontal gyri were significantly active when the participants concealed their true identities. In addition, significant activation differences were also found in the right insular cortex between the identity concealment condition and the control condition. Several existing studies have suggested the activation in the insula to be related to self-awareness [3], [55], [56]. It is possible that the activation of this particular brain region in the present study might be due to the fact that the participants automatically responded to their own names even though they behaviorally tried to suppress their responses to such personally significant information. In contrast, the identity faking condition, where participants had to assume a name that was not their own, produced significantly greater activations in the right SFG, an area that has been suggested to be associated with self-consciousness [13].

However, the existing research on the processing of information concerning the self has consistently revealed the involvement of the cortical midline structures [8], [51]. However, we failed to obtain any significant activations in such structures in the identity concealment nor faking conditions. Uddin and colleagues (2007) suggested that the right frontoparietal system is involved in representing the physical, embodied self, whereas the cortical midline structures seem to be more involved in maintaining a self-representation in evaluative terms. In our identity concealment condition, participants were asked to deny their own name, and in the identity faking condition, they were asked to admit that a new name was their own. Both tasks were name recognition tasks that were not evaluative and thus might be too neutral to engage the self-evaluative regions. In addition, the present paradigm involves strong cognitive functions which may have spoiled the involvement of midline regions.

### Identity Faking and Concealment in the Deception Network

Consistent with our hypothesis, in the identity concealment condition, significant activation differences were found in the inferior frontal gyrus between the concealed name (participant’s true name) and the control name. The inferior frontal gyrus was found to play an important role in inhibitory control [16], [57]. In the identity concealment condition, people were required to inhibit admitting their own name. In addition to the other types of deception [16], our results confirmed that inhibitory control was involved in the process of identity concealment.

Significant activation differences were found in the superior frontal gyrus/DLPFC between identity faking and the control condition. Also, from the ROI analysis, we found that identity faking elicited a significantly larger percentage signal increase than the identity concealment condition in the same area. The DLPFC has been implicated in maintenance of information [58]. Because participants were asked to suppress their true name and assume another name, they had to constantly keep in mind what their fake name was, which is a taxing task in terms of working memory. Thus, the significant activation difference in the SFG might be related to the working memory demand in the identity faking condition.

Our findings regarding the involvement of the IFG and SFG in identity concealment and faking respectively, suggested that although both require the execution of a response that is incompatible with the truth, the different cognitive components of executive controls may play different roles in the process of identity concealment and faking: identity concealment is related with inhibition control while identity faking requires the involvement of working memory.

Inconsistent with other neuroimaging studies on deception [23]–[25], [31], [36], [40], we did not find significant activations in the ACC in both the identity concealment and faking conditions. However, our results were consistent with another set of studies [26], [52], demonstrating that ACC activations may be moderated by task difficulty. In the present study, the task for participants was rather simple, as they only needed to either assume a fake name or deny that their real name was their own. Had we used more cognitively demanding deception tasks, significant differences may have been found between the experimental conditions and the control condition. Indeed, existing studies using the Guilt Knowledge Task revealed the important role of the ACC [59], [60]. The GKT task is a far more cognitively demanding task than our tasks. In that task, participants were repeatedly asked a series of questions about different aspects of an event. Participants not only had to lie about them but also had to keep track of their responses, such that their responses would be consistent with each other. Perhaps for this reason, significant activations were found in the ACC in such studies.

### Identity Faking vs. Concealment Condition

Simple GLM contrasts showed that there were no significant differences between the identity concealment condition and the faking condition in any brain regions. However, the more sensitive non-linear SVM machine learning analysis reliably differentiated the brain activities in the six ROIs associated with either identity faking or identity concealment. Furthermore, the SVM analysis on the data from each ROI (the right IFG, right insula, right SFG, right caudate, and left calcarine) also independently differentiated the brain activities between the identity faking and concealment conditions. This finding suggests that a non-linear analysis may be a useful tool for detecting complex neural response patterns to reveal subtle differences between experimental conditions as opposed to the traditoinal voxel based linear analysis, which is too insensitive to reveal subtle differences [61].

It is worth noting that the differences between the identity concealment and faking conditions was similar to the differences between pretending not to know and pretending to know, which were found in previous studies [17], [31]. Abe et al. (2008) asked participants to tell truths and lies about true targets or new targets, and compared the difference of neural responses between pretending not to know and pretending to know. Although statistical comparisons were not made, the results provided by the authors clearly showed the IFG to be more active during the pretending not to know than during the pretending to know. Consistent with their findings, we found that the IFG was greater in the identity concealment condition than in the identity faking condition.

In summary, the present study used fMRI methodology to examine the neural correlates of two different types of identity conflict: identity faking and concealment. We found that both the identity concealment and faking conditions engaged the neural network for the processing of information concerning the self and the deception network. Also, although identity faking and concealment engaged some common brain regions, the neural networks for the two types of identity conflict were clearly distinct. Identity concealment activated the inferior frontal gyrus and right insula, whereas identity faking activated the superior frontal gyrus. In addition, the support vector machine learning method was shown to be a useful method to differentiate subtle differences in activation patterns between identity concealment and faking.

## References

[1] Erikson EH (1950) Childhood and society. New York: Norton.

[2] Decety J, Sommerville JA (2003) Shared representations between self and other: A social cognitive neuroscience view. Trends in Cognitive Sciences 7: 527–533. 14643368 10.1016/j.tics.2003.10.004

[3] Devue C, Collette F, Balteau E, Degueldre C, Luxen A, et al (2007) Here I am: The cortical correlates of visual self-recognition. Brain Research 1143: 169–182. 17306235 10.1016/j.brainres.2007.01.055

[4] Heatherton TF, Macrae CN, Kelley WM (2004) What the social brain sciences can tell us about the self. Current Directions in Psychological Science 13: 190–193.

[5] Kaplan JT, Aziz-Zadeh L, Uddin LQ, Iacoboni M (2008) The self across the senses: An fMRI study of self-face and self-voice recognition. Social Cognitive and Affective Neuroscience 3: 218–223. 19015113 10.1093/scan/nsn014 PMC2566765

[6] Northoff G, Heinzel A, de Greck M, Bermpohl F, Dobrowolny H, et al (2006) Self-referential processing in our brain–A meta-analysis of imaging studies on the self. Neuroimage 31: 440–457. 16466680 10.1016/j.neuroimage.2005.12.002

[7] Platek SM, Keenan JP, Gallup GG (2004) Where am I? The neurological correlates of self and other. Cognitive Brain Research 19: 114–122. 15019708 10.1016/j.cogbrainres.2003.11.014

[8] Uddin LQ, Iacoboni M, Lange C, Keenan JP (2007) The self and social cognition: The role of cortical midline structures and mirror neurons. Trends in Cognitive Sciences 11: 153–157. 17300981 10.1016/j.tics.2007.01.001

[9] Platek SM, Wathne K, Tierney NG, Thomson JW (2008) Neural correlates of self-face recognition: An effect-location meta-analysis. Brain Research 1232: 173–184. 18656465 10.1016/j.brainres.2008.07.010

[10] Uddin LQ, Kaplan JT, Molnar-Szakacs I, Zaidel E, Iacoboni M (2005) Self-face recognition activates a frontoparietal “mirror” network in the right hemisphere:an event-related fMRI study. Neuroimage 25: 926–935. 15808992 10.1016/j.neuroimage.2004.12.018

[11] Perrin F, Maquet P, Peigneux P, Ruby P, Degueldre C, et al (2005) Neural mechanisms involved in the detection of our first name: A combined ERPs and PET study. Neuropsychologia 43: 12–19. 15488900 10.1016/j.neuropsychologia.2004.07.002

[12] Carmody DP, Lewis M (2006) Brain activation when hearing one’s own and others’ names. Brain Research 1116: 153–158. 16959226 10.1016/j.brainres.2006.07.121 PMC1647299

[13] Kampe KKW, Frith CD, Frith U (2003) “Hey John”: Signals conveying communicative intention toward the self activate brain regions associated with“ mentalizing,” regardless of modality. Journal of Neuroscience 23: 5258–5263. 12832550 10.1523/JNEUROSCI.23-12-05258.2003 PMC6741156

[14] Tacikowski P, Brechmann A, Marchewka A, Jednoróg K, Dobrowolny M, et al (2010) Is it about the self or the significance? An fMRI study of self-name recognition. Social Neuroscience 6: 1–10. 20602286 10.1080/17470919.2010.490665

[15] Abe N (2011) How the brain shapes deception: An integrated review of the literature. The Neuroscientist 17: 560–574. 21454323 10.1177/1073858410393359

[16] Christ SE, Van Essen DC, Watson JM, Brubaker LE, McDermott KB (2009) The contributions of prefrontal cortex and executive control to deception: Evidence from activation likelihood estimate meta-analyses. Cerebral Cortex 19: 1557–1566. 18980948 10.1093/cercor/bhn189 PMC2693617

[17] Abe N, Okuda J, Suzuki M, Sasaki H, Matsuda T, et al (2008) Neural correlates of true memory, false memory, and deception. Cerebral Cortex 18: 2811–2819. 18372290 10.1093/cercor/bhn037 PMC2583150

[18] Lee TMC, Liu HL, Tan LH, Chan CCH, Mahankali S, et al (2002) Lie detection by functional magnetic resonance imaging. Human Brain Mapping 15: 157–164. 11835606 10.1002/hbm.10020 PMC6872015

[19] Lee T, Liu HL, Chan CCH, Ng YB, Fox PT, et al (2005) Neural correlates of feigned memory impairment. Neuroimage 28: 305–313. 16165373 10.1016/j.neuroimage.2005.06.051

[20] Lee TMC, Au RKC, Liu H-L, Ting KH, Huang C-M, et al (2009) Are errors differentiable from deceptive responses when feigning memory impairment? An fMRI study. Brain and Cognition 69: 406–412. 18938008 10.1016/j.bandc.2008.09.002

[21] Davatzikos C, Ruparel K, Fan Y, Shen DG, Acharyya M, et al (2005) Classifying spatial patterns of brain activity with machine learning methods: Application to lie detection. Neuroimage 28: 663–668. 16169252 10.1016/j.neuroimage.2005.08.009

[22] Gamer M, Klimecki O, Bauermann T, Stoeter P, Vossel G (2009) fMRI-activation patterns in the detection of concealed information rely on memory-related effects. Social Cognitive and Affective Neuroscience doi:10.109. 10.1093/scan/nsp005 PMC3375883 19258375

[23] Kozel FA, Johnson KA, Mu Q, Grenesko EL, Laken SJ, et al (2005) Detecting deception using functional magnetic resonance imaging. Biological Psychiatry 58: 605–613. 16185668 10.1016/j.biopsych.2005.07.040

[24] Langleben DD, Schroeder L, Maldjian JA, Gur RC, McDonald S, et al (2002) Brain activity during simulated deception: An event-related functional magnetic resonance study. Neuroimage 15: 727–732. 11848716 10.1006/nimg.2001.1003

[25] Langleben DD, Loughead JW, Bilker WB, Ruparel K, Childress AR, et al (2005) Telling truth from lie in individual subjects with fast event-related fMRI. Human Brain Mapping 26: 262–272. 16161128 10.1002/hbm.20191 PMC6871667

[26] Luan Phan K, Magalhaes A, Ziemlewicz TJ, Fitzgerald DA, Green C, et al (2005) Neural correlates of telling lies: A functional magnetic resonance imaging study at 4 Tesla. Academic Radiology 12: 164–172. 15721593 10.1016/j.acra.2004.11.023

[27] Priori A, Mameli F, Cogiamanian F, Marceglia S, Tiriticco M, et al (2008) Lie-specific involvement of dorsolateral prefrontal cortex in deception. Cerebral Cortex 18: 451–455. 17584853 10.1093/cercor/bhm088

[28] Ito H, Yamauchi H, Kaneko H, Yoshikawa T, Nomura K, et al (2011) Prefrontal overactivation, autonomic arousal, and task performance under evaluative pressure: A near-infrared spectroscopy (NIRS) study. Psychophysiology 48: 1563–1571. 21564124 10.1111/j.1469-8986.2011.01220.x

[29] Lee TMC, Lee TMY, Raine A, Chan CCH (2010) Lying about the valence of affective pictures: An fMRI study. PLoS ONE 5: e12291. 20811624 10.1371/journal.pone.0012291 PMC2928271

[30] Bhatt S, Mbwana J, Adeyemo A, Sawyer A, Hailu A, et al (2009) Lying about facial recognition: An fMRI study. Brain and Cognition 69: 382–390. 18848742 10.1016/j.bandc.2008.08.033

[31] Abe N, Suzuki M, Tsukiura T, Mori E, Yamaguchi K, et al (2006) Dissociable roles of prefrontal and anterior cingulate cortices in deception. Cerebral Cortex 16: 192–199. 15858160 10.1093/cercor/bhi097

[32] Abe N, Suzuki M, Mori E, Itoh M, Fujii T (2007) Deceiving others: Distinct neural responses of the prefrontal cortex and amygdala in simple fabrication and deception with social interactions. Journal of Cognitive Neuroscience 19: 287–295. 17280517 10.1162/jocn.2007.19.2.287

[33] Ganis G, Kosslyn SM, Stose S, Thompson WL, Yurgelun-Todd DA (2003) Neural correlates of different types of deception: An fMRI investigation. Cerebral Cortex 13: 830–836. 12853369 10.1093/cercor/13.8.830

[34] Grezes J, Berthoz S, Passingham RE (2006) Amygdala activation when one is the target of deceit: Did he lie to you or to someone else? Neuroimage 30: 601–608. 16257239 10.1016/j.neuroimage.2005.09.038

[35] Grezes J, Frith C, Passingham RE (2004) Brain mechanisms for inferring deceit in the actions of others. Journal of Neuroscience 24: 5500–5505. 15201322 10.1523/JNEUROSCI.0219-04.2004 PMC6729335

[36] Nuñez JM, Casey BJ, Egner T, Hare T, Hirsch J (2005) Intentional false responding shares neural substrates with response conflict and cognitive control. Neuroimage 25: 267–277. 15734361 10.1016/j.neuroimage.2004.10.041

[37] Spence SA, Kaylor-Hughes C, Farrow TFD, Wilkinson ID (2008) Speaking of secrets and lies: The contribution of ventrolateral prefrontal cortex to vocal deception. Neuroimage 40: 1411–1418. 18308586 10.1016/j.neuroimage.2008.01.035

[38] Ganis G, Morris RR, Kosslyn SM (2009) Neural processes underlying self-and other-related lies: An individual difference approach using fMRI. Social Neuroscience 4: 539–553. 18925536 10.1080/17470910801928271

[39] Greene JD, Paxton JM (2009) Patterns of neural activity associated with honest and dishonest moral decisions. Proceedings of the National Academy of Sciences 106: 12506–12511. 10.1073/pnas.0900152106 PMC2718383 19622733

[40] Kozel FA, Padgett TM, George MS (2004) A replication study of the neural correlates of deception. Behavioral Neuroscience 118: 852–856. 15301611 10.1037/0735-7044.118.4.852

[41] Langleben DD (2008) Detection of deception with fMRI: Are we there yet? Legal and Criminological Psychology 13: 1–9.

[42] Sip KE, Roepstorff A, McGregor W, Frith CD (2008) Detecting deception: the scope and limits. Trends in cognitive sciences 12: 48–53. 18178516 10.1016/j.tics.2007.11.008

[43] Vrij A, Fisher R, Mann S, Leal S (2006) Detecting deception by manipulating cognitive load. Trends in Cognitive Sciences 10: 141–142. 16516533 10.1016/j.tics.2006.02.003

[44] Gombos VA (2006) The cognition of deception: The role of executive processes in producing lies. Genetic, Social, and General Psychology Monographs 132: 197–214. 10.3200/mono.132.3.197-214 17969998

[45] Johnson R, Barnhardt J, Zhu J (2004) The contribution of executive processes to deceptive responding. Neuropsychologia 42: 878–901. 14998703 10.1016/j.neuropsychologia.2003.12.005

[46] Talwar V, Lee K (2008) Social and cognitive correlates of children’s lying behavior. Child Development 79: 866–881. 18717895 10.1111/j.1467-8624.2008.01164.x PMC3483871

[47] Miyake A, Friedman NP, Emerson MJ, Witzki AH, Howerter A, et al (2000) The unity and diversity of executive functions and their contributions to complex “frontal lobe” tasks: A latent variable analysis. Cognitive Psychology 41: 49–100. 10945922 10.1006/cogp.1999.0734

[48] Khatib SM (1995) Personal names and name changes. Journal of Black Studies 25: 349–353.

[49] Watson RS (1986) The named and the nameless: gender and person in Chinese society. American Ethnologist 13: 619–631.

[50] Sugiura M, Watanabe J, Maeda Y, Matsue Y, Fukuda H, et al (2005) Cortical mechanisms of visual self-recognition. Neuroimage 24: 143–149. 15588605 10.1016/j.neuroimage.2004.07.063

[51] Northoff G, Bermpohl F (2004) Cortical midline structures and the self. Trends in Cognitive Sciences 8: 102–107. 15301749 10.1016/j.tics.2004.01.004

[52] Gamer M, Bauermann T, Stoeter P, Vossel G (2007) Covariations among fMRI, skin conductance, and behavioral data during processing of concealed information. Human Brain Mapping 28: 1287–1301. 17290371 10.1002/hbm.20343 PMC6871443

[53] Chang C, Lin C (2011) LIBSVM: A library for support vector machines. ACM Transactions on Intelligent Systems and Technology 2: 1–27.

[54] Bishop CM (2007) Pattern recognition and machine learning. Springer.

[55] Morita T, Itakura S, Saito DN, Nakashita S, Harada T, et al (2008) The role of the right prefrontal cortex in self-evaluation of the face: A functional magnetic resonance imaging study. Journal of Cognitive Neuroscience 20: 342–355. 18275339 10.1162/jocn.2008.20024

[56] Sperduti M, Delaveau P, Fossati P, Nadel J (2011) Different brain structures related to self- and external-agency attribution: a brief review and meta-analysis. Brain structure & function 216: 151–157. 21212978 10.1007/s00429-010-0298-1

[57] Aron AR, Robbins TW, Poldrack RA (2004) Inhibition and the right inferior frontal cortex. Trends in Cognitive Sciences 8: 170–177. 15050513 10.1016/j.tics.2004.02.010

[58] D’Esposito M, Postle BR, Rypma B (2000) Prefrontal cortical contributions to working memory: Evidence from event-related fMRI studies. Experimental Brain Research 133: 3–11. 10933205 10.1007/s002210000395

[59] Rosenfeld JP, Biroschak JR, Furedy JJ (2006) P300-based detection of concealed autobiographical versus incidentally acquired information in target and non-target paradigms. International Journal of Psychophysiology 60: 251–259. 16137781 10.1016/j.ijpsycho.2005.06.002

[60] Seymour T, Seifert C (2000) Using response time measures to assess “guilty knowledge”. Journal of Applied Psychology 85: 30–37. 10740954 10.1037/0021-9010.85.1.30

[61] Lao Z, Shen D, Xue Z, Karacali B, Resnick SM, et al (2004) Morphological classification of brains via high-dimensional shape transformations and machine learning methods. NeuroImage 21: 46–57. 14741641 10.1016/j.neuroimage.2003.09.027

